# A Metabolomic Analysis of Two Intravenous Lipid Emulsions in a Murine Model

**DOI:** 10.1371/journal.pone.0059653

**Published:** 2013-04-02

**Authors:** Brian T. Kalish, Hau D. Le, Kathleen M. Gura, Bruce R. Bistrian, Mark Puder

**Affiliations:** 1 Department of Surgery and The Vascular Biology Program, Boston Children’s Hospital, Boston, Massachusetts, United States of America; 2 Department of Pharmacy, Boston Children’s Hospital, Boston, Massachusetts, United States of America; 3 Department of Medicine, Beth Israel Deaconess Medical Center, Boston, Massachusetts, United States of America; 4 Department of Surgery, Beth Israel Deaconess Medical Center, Boston, Massachusetts, United States of America; Bambino Gesu’ Children Hospital, Italy

## Abstract

**Background:**

Parenteral nutrition (PN), including intravenous lipid administration, is a life-saving therapy but can be complicated by cholestasis and liver disease. The administration of intravenous soy bean oil (SO) has been associated with the development of liver disease, while the administration of intravenous fish oil (FO) has been associated with the resolution of liver disease. The biochemical mechanism of this differential effect is unclear. This study compares SO and FO lipid emulsions in a murine model of hepatic steatosis, one of the first hits in PN-associated liver disease.

**Methods:**

We established a murine model of hepatic steatosis in which liver injury is induced by orally feeding mice a PN solution. C57BL/6J mice were randomized to receive PN alone (a high carbohydrate diet (HCD)), PN plus intravenous FO (Omegaven®; Fresenius Kabi AG, Bad Homburg VDH, Germany), PN plus intravenous SO (Intralipid®; Fresenius Kabi AG, Bad Homburg v.d.H., Germany, for Baxter Healthcare, Deerfield, IL), or a chow diet. After 19 days, liver tissue was harvested from all animals and subjected to metabolomic profiling.

**Results:**

The administration of an oral HCD without lipid induced profound hepatic steatosis. SO was associated with macro- and microvesicular hepatic steatosis, while FO largely prevented the development of steatosis. 321 detectable compounds were identified in the metabolomic analysis. HCD induced *de novo* fatty acid synthesis and oxidative stress. Both FO and SO relieved some of the metabolic shift towards *de novo* lipogenesis, but FO offered additional advantages in terms of lipid peroxidation and the generation of inflammatory precursors.

**Conclusions:**

Improved lipid metabolism combined with reduced oxidative stress may explain the protective effect offered by intravenous FO *in vivo*.

## Introduction

Parenteral nutrition (PN) is a life-saving therapy for many patients with a variety of intestinal failure syndromes. However, PN is associated with a severe form of hepatobiliary dysfunction: PN-associated liver disease (PNALD). The manifestations of PNALD can range from steatosis and cholestasis to hepatic fibrosis, cirrhosis, and end-stage liver disease. [1], While adults with PNALD most commonly present only with hepatic steatosis, neonates and infants most often experience cholestasis that can rapidly progress to end-stage liver disease without treatment.

PNALD is estimated to affect as many as 85% of neonates and 60% of infants who are PN-dependent for their nutritional needs. [1] In a study of 272 infants with intestinal failure, mortality and rate of intestinal transplantation at 3 years was 27% and 26%, respectively. [2].

PN is administered in combination with a lipid emulsion to provide additional non-protein calories and prevent essential fatty acid deficiency (EFAD). There is growing evidence that PNALD may be, in part, due to the composition of the lipid emulsion that is administered with PN. [3], [4] Currently, the only FDA approved lipid emulsions in the United States are comprised of safflower oil, soybean oil, or a combination of the two, although effectively only the soybean oil emulsion is now available for clinical use. These lipid emulsions are rich in ω-6 polyunsaturated fatty acids, which are the parent molecules in the synthesis of many pro-inflammatory bioactive compounds. Other lipid emulsions available in Europe and elsewhere contain a minimal amount of fish oil. FO contains high concentrations ω-3 polyunsaturated fatty acids, which are parent molecules to largely anti-inflammatory, pro-resolving mediators.

Clinically, the administration of FO-based lipid emulsions has shown promise in the prevention and reversal of PNALD. Puder and colleagues have reported that infants on FO reversed cholestasis six times faster than infants on SO. [5] The molecular mechanisms underlying the clinical benefit observed with FO are unclear. The etiology of hepatobiliary dysfunction and systemic inflammation is likely multifactorial, with the relative effects of enteral versus parenteral feeding, high-carbohydrate and high-glucose nutrition, and the composition of intravenous lipid being poorly understood. The goal of this study was to characterize the simultaneous, global metabolic changes associated with the oral administration of a PN solution alone or in combination with the intravenous administration of either FO or SO.

The use of a broad metabolomics platform provides the distinct advantage of analyzing dozens of metabolic pathways simultaneously, thereby allowing a systems-level approach to the complex interplay among metabolic processes. We proposed several hypotheses: (1) A high-carbohydrate, PN solution provided without lipid would demonstrate a marked increase in *de novo* fatty acid synthesis and oxidative stress; (2) Both FO and SO would offer some relief from these effects of exclusive carbohydrate provision seen with the HCD alone; and (3) FO would further decrease *de novo* lipogenesis, lipid peroxidation, and inflammatory precursors relative to SO.

## Materials and Methods

### Ethics Statement

Animal protocols complied with the NIH Animal Research Advisory Committee guidelines and were approved by the Children’s Hospital Boston Animal Care and Use Committee.

### Mouse Model of Hepatic Steatosis

We established a murine model of hepatic steatosis in which liver injury is induced by orally feeding mice a PN solution. Male 6-week-old C57BL6/J mice (Jackson Laboratories, Bar Harbor, ME) were randomized to four groups, as previously described. [6] Group 1 received a standard chow diet (Prolab Isopro, RMH 3000 #25; containing 14% fat, 26% protein, and 60% carbohydrate by calories; Prolabs Purina, Richmond, IN). Groups 2–4 were placed on an exclusive ad libitum liquid, fat-free, high-carbohydrate diet (HCD). This solution was identical to typical PN solutions used at Boston Children’s Hospital. It contained 20% dextrose, a mixture of 2% essential and nonessential amino acids (Trophamine, B. Braun Medical, Irvine, Calif), 0.2% pediatric trace elements (American Reagent, Shirley, NY), pediatric multivitamins (Hospira, Clayton, NC), 30 mEq of sodium, 20 mEq of potassium, 15 mEq of calcium, 10 mEq of magnesium, 10 mmol of phosphate, 36.67 mEq of chloride, and 19.4 mEq of acetate per liter. This liquid diet was placed in a single bottle per cage, and replaced daily to avoid contamination. Mice received no other source of nutrition or hydration.

Group 2 received the HCD plus intravenous normal saline (designated “HCD” hereafter). Groups 3 and 4 received commercial lipid emulsions via tail vein injections every other day (2.4 g of fat/kg body weight per day). Group 3 received the HCD plus intravenous fish oil (FO) (Omegaven®; Fresenius Kabi AG, Bad Homburg VDH, Germany); Group 4 received the HCD plus intravenous soy bean oil (SO) (Intralipid®; Fresenius Kabi AG, Bad Homburg v.d.H., Germany, for Baxter Healthcare, Deerfield, IL). Table 1 lists the composition of these commercial lipid emulsions.

**Table 1:** Composition of commercial lipid emulsions.

Lipid Emulsions	Intralipid®	Omegaven®
Oil source (g)		
Soy bean	10	0
Fish oil	0	10
α-tocopherol (mg/L)	38	150–296
Phytosterols (mg/K)	348±33	0
Fat composition (g)		
Linolenic	5.0	0.1–0.7
α-Linolenic	0.9	<0.2
EPA	0	1.28–2.82
DHA	0	1.44–3.09
Oleic	2.6	0.6–1.3
Palmitic	1.0	0.25–1
Stearic	0.35	0.05–0.2
Arachidonic	0	0.1–0.4

Mice were housed five per wire-bottom cage in a barrier room with regulated temperature (21°C±1.6°C), humidity (45%±10%), and an alternating 12-hour light and dark cycle. At 19 days, the animals were euthanized with carbon dioxide inhalation, after which the liver was harvested for histology and metabolic profiling.

### Histology

A designated lobe of the liver was fixed in 10% formalin overnight, washed with chilled phosphate buffered saline solution, and embedded in paraffin. The sections were stained with hematoxylin and eosin to examine cellular architecture. Another portion of the liver was collected as frozen sections, placed in embedding medium (Optimal Cutting Temperature OCT; Sakura Fenetek, Torrance, CA), and promptly immersed in liquid nitrogen. Sections were stained at the Department of Pathology, Children’s Hospital Boston with Oil Red “O” for hepatic fat. A last portion was immediately snap-frozen in liquid nitrogen and placed on dry ice for future metabolomic analysis. The samples were stored at −80°C.

### Metabolomic Analysis

The sample preparation process was carried out using the automated MicroLab STAR® system from Hamilton Company. Recovery standards were added prior to the first step in the extraction process for QC purposes. Sample preparation was conducted using a proprietary series of organic and aqueous extractions to remove the protein fraction while allowing maximum recovery of small molecules. The resulting extract was divided into two fractions: one for analysis by LC and one for analysis by GC. Samples were placed briefly on a TurboVap® (Zymark, Hopkington, MA) to remove the organic solvent. Each sample was then frozen and dried under a vacuum. Samples were then prepared for the appropriate instrument, either LC/MS or GC/MS.

The LC/MS portion of the platform was based on a Waters ACQUITY UPLC and a Thermo-Finnigan LTQ mass spectrometer, which consisted of an electrospray ionization (ESI) source and linear ion-trap (LIT) mass analyzer. The samples destined for GC/MS analysis were re-dried under vacuum desiccation for a minimum of 24 hours prior to being derivatized under dried nitrogen using bistrimethyl-silyl-triflouroacetamide (BSTFA). The GC column was 5% phenyl and the temperature ramp was from 40° to 300°C in a 16 minute period. Samples were analyzed on a Thermo-Finnigan Trace DSQ fast-scanning single-quadrupole mass spectrometer using electron impact ionization.

The raw data was extracted, peak-identified and QC processed. Compounds were identified by comparison to library entries of purified standards or recurrent unknown entities. This library contains the retention time/index (RI), mass to charge ratio (m/z), and chromatographic data (including MS/MS spectral data) on all molecules present in the analysis. Furthermore, biochemical identifications were based on three criteria: retention index within a narrow RI window of the proposed identification, nominal mass match to the library +/−0.2 amu, and the MS/MS forward and reverse scores between the experimental data and authentic standards. The MS/MS scores were based on a comparison of the ions present in the experimental spectrum to the ions present in the library spectrum. While there may be similarities between these molecules based on one of these factors, the use of all three data points can be utilized to distinguish and differentiate biochemicals. More than 2400 commercially available purified standard compounds have been acquired and registered into LIMS for distribution to both the LC and GC platforms for determination of their analytical characteristics.

**Figure 1:**
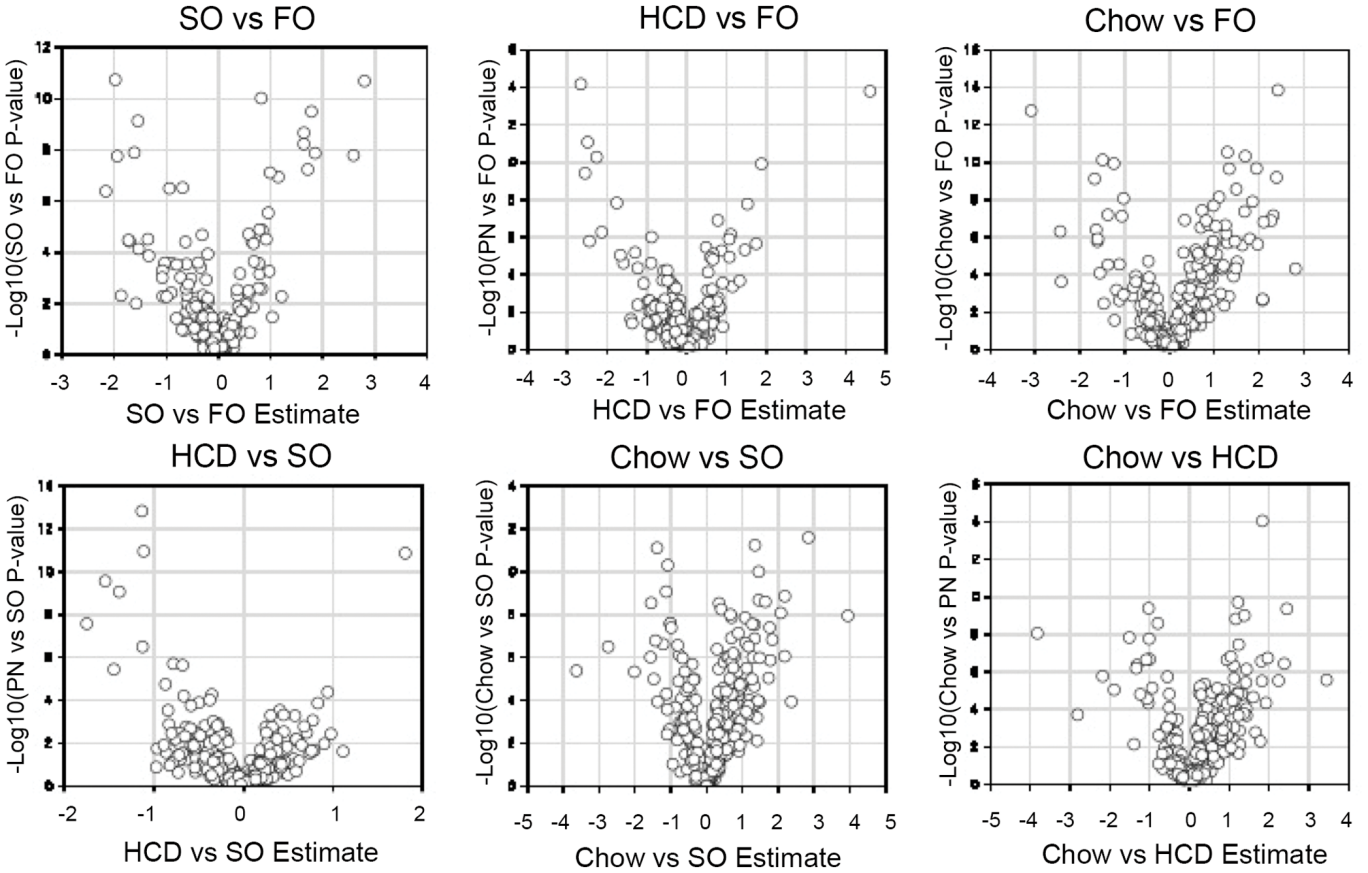
Volcano plots.

Missing values (if any) were assumed to be below the level of detection. However, biochemicals that were detected in all samples from one or more groups but not in samples from other groups were assumed to be near the lower limit of detection in the groups in which they were not detected. In this case, the lowest detected level of these biochemicals was imputed for samples in which that biochemical was not detected. Following log transformation and imputation with minimum observed values for each compound, Welch’s two-sample t-test was used to identify biochemicals that differed significantly between experimental groups. A p-value of 0.05 was adapted as the significant level. q-values were provide but not used for significance. Volcano plots were constructed to visualize the distribution of p-values (Figure 1). Pathways were assigned for each metabolite, allowing examination of overrepresented pathways. All of the statistical analyses were carried out using Array Studio v5.0.

## Results

### Histology

**Figure 2:**
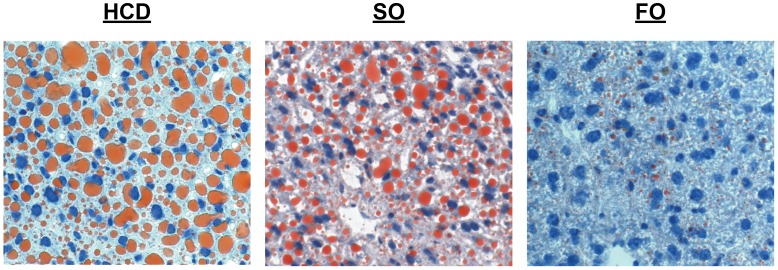
Representative Images of Liver Tissue Stained with Oil Red O.

As previously described, the administration of an oral HCD without lipid induced profound hepatic steatosis, consistent with both a high glucose diet and EFAD (Figure 2A). SO induced macro- and microvesicular hepatic steatosis (Figure 2B), while FO largely prevented the development of steatosis (Figure 2C).

### Metabolomic Analysis

Three hundred and twenty one detectable compounds were identified in the metabolomic analysis.

#### Essential fatty acids and their derivatives (Figure 3)

The essential fatty acids – classically defined as linoleate (linoleic acid) and linolenate (linolenic acid) – are the fatty acids that cannot be synthesized *de novo* in humans. In this analysis, α-linolenic acid could not be distinguished from γ-linolenic acid. As expected, the levels of linoleate and linolenate decreased significantly in the group receiving HCD alone compared to chow (0.63 and 0.60 respectively, p<0.05). Similar decreases were observed for *e*icosapentaenoic acid (EPA) and docosapentaenoic acid (DPA) – derived from ALA (EPA) and LA(DPA) – in HCD compared to chow (0.32 and 0.46 respectively, p<0.05). SO increased most major fatty acids, including dihomo-gammalinolenate (2.14, p<0.05) derived from LA, relative to the EFA-deficient HCD alone group. Notably, SO did not increase levels of the omega-3 fatty acids docosahexaenoic Acid (DHA) and DPA, even though SO contains substantial amounts (about 7%) of ALA. Compared to SO, FO increased the ω-3 FAs – DPA (5.18) and DHA (2.61) – and decreased the ω-6 FAs – LA (0.20) and arachidonic acid (0.44) (p<0.05).

**Figure 3:**
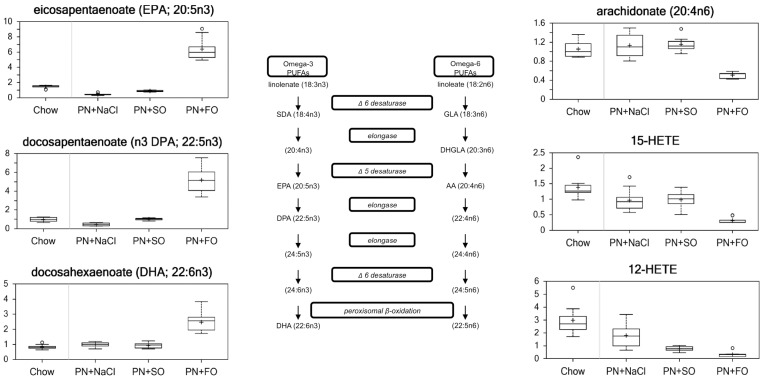
Metabolites PUFAs and their derivatives.

The hydroxyeicosatetraenoic acids (HETEs) are major mono-hydroxylated arachidonic acid metabolites that are produced during inflammatory and immunological responses. Compared to SO, FO decreased 12-HETE (0.42) and 15-HETE (0.32) (p<0.05).

#### Oxidative stress (figures 4 and 5)

Glutathione metabolism is one of the major endogenous defense systems to combat cellular oxidative stress. Relative to chow, HCD alone decreased levels of 5-oxoproline (0.72) and most of the detected γ–glutamyl amino acids: γ-glutamylvaline (0.54), γ-glutamylglutamate (0.80), γ-glutamylphenylalanine (0.54), γ-glutamylthreonine (0.26) (p<0.05), all of which suggests slower glutathione turnover in the HCD group. Compared to chow, the relative concentration of taurine in the HCD group was 1.35 (p<0.05).

**Figure 4:**
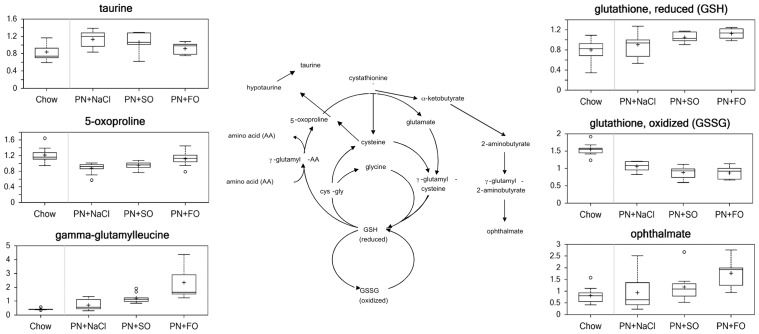
Oxidative Stress Metabolites.

**Figure 5:**
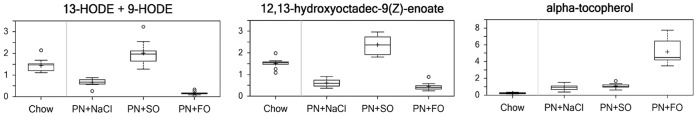
Lipid Peroxidation and Anti-oxidant Metabolites.

Both FO and SO increased the concentration of reduced glutathione relative to chow (1.41 and 1.31 respectively; p<0.05). Relative to HCD, FO increased the concentration of reduced glutathione (1.24; p<0.05); the trend was that SO increased reduced glutathione relative to HCD (1.16), but the difference was not statistically significant. Both FO and SO decreased the concentration of oxidized glutathione (GSSG) compared to chow (0.56 and 0.57 respectively; p<0.05) and compared to HCD (0.82 and 0.83 respectively; p<0.05).

FO decreased taurine relative to HCD (0.81; p<0.05), suggesting less diversion of cysteine from glutathione synthesis. Compared to SO, the relative concentrations of 5-oxoproline and opthalmate were higher in FO (1.17 and 1.50; p<0.05).

9-HODE and 13-HODE are the oxidized products of linoleic acid. Relative to chow, both HCD (0.46) and FO (0.11) decreased these metabolites (p<0.05). However, SO increased the concentration of 9-HODE and 13-HODE relative to chow (1.40; p<0.05). Compared to HCD, SO increased the concentration of this metabolite (3.05; p<0.05), while FO decreased its concentration (0.25; p<0.05). FO dramatically decreased the concentration of these oxidation products relative to SO (0.08; p<0.05).

12,13-hydroxyoctadec-9(Z)-enoate is a lipid peroxidation product that has neutrophil chemotactic properties. Relative to chow, both HCD (0.40) and FO (0.30) decreased this metabolite (p<0.05), while SO increased its concentration (1.57; p<0.05). Compared to HCD, SO increased the concentration of this metabolite (3.93), and FO trended towards a decrease in concentration (0.74), though it did not reach statistical significance. FO lowered the concentration of 12,13-hydroxyoctadec-9(Z)-enoate (0.19) as compared to SO (p<0.05).

α-tocopherol is a form of Vitamin E that acts as an antioxidant in the glutathione peroxidase pathway. Compared to chow, α-tocopherol was increased in HCD (4.65) and SO (3.95), but much more dramatically in FO (21.89)(p<0.05). There was no difference between SO and HCD in terms of α-tocopherol, but FO increased α-tocopherol relative to both HCD (5.55) and SO (4.70)(p<0.05).

#### Fatty acid synthesis (figure 6)

The formation of malonyl-CoA is the committed step in FA biosynthesis. The level of malonylcarnitine normally reflects the level of malonyl-CoA. Compared to chow, both HCD (0.33) and SO (0.41) decreased the relative concentration of malonylcarnitine (p<0.05), while FO produced the concentration closest to that of chow (0.78). Compared to HCD, FO (2.36) but not SO (1.25) produced a statistically significant increase in malonylcarnitine (p<0.05). FO significantly increased malonylcarnitine relative to SO (1.89; p<0.05).

**Figure 6:**
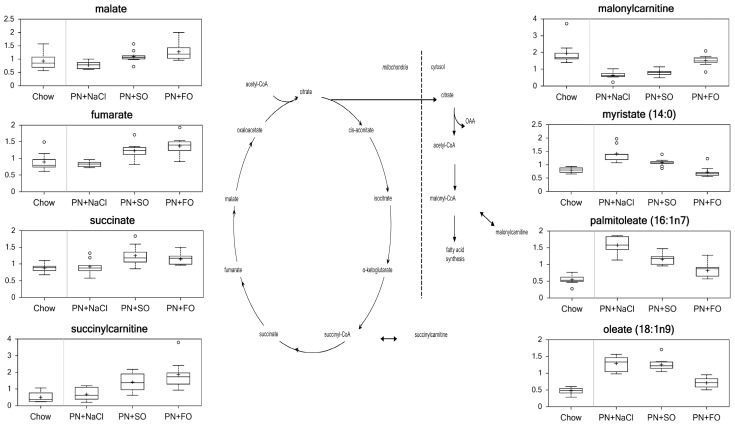
Metabolites of Fatty Acid Synthesis and TCA Cycle.

The levels of long-chain fatty acids (LCFAs), including myristate, myristoleate, palmitate, palmitoleate, 10-heptadecenoate, oleate, 10-nonadecenoate, eicosenoate, dihomo-linoleate, docosadienoate, and docosatrienoate were increased in HCD as compared to chow (p<0.05), suggesting greater *de novo* lipogenesis particularly as reflected in the increased saturated fatty acids and the monounsaturated fatty acids with an ω-9 configuration. The glycogen breakdown products maltotriose, maltotetraose, maltopentaose, and maltohexaose also decreased significantly in the HCD group compared to chow (p<0.05), consistent with reduced glyogenolysis.

The intermediates in the TCA cycle – malate, fumarate, succinate and succinylcarnitine – did not differ between the HCD and chow groups. These intermediates increased significantly in both SO and FO groups in relation to HCD and chow (p<0.05), which suggests improved aerobic glycolysis with the addition of lipid of either type.

Carnitine transports long-chain acyl groups from fatty acids into the mitochondrial matrix, where they can be metabolized via beta-oxidation. Compared to the chow group, carnitine was reduced significantly in HCD (0.67) and SO (0.74), but there was no statistically significant difference between FO and chow. Relative to HCD, FO had a higher concentration of carnitine (1.38; p<0.05), while SO did not. FO had a higher concentration of carnitine compared to SO (1.25; p<0.05), which is indicative of increased fatty acid oxidation with FO. The levels of ascorbate were increased in both FO (1.25) and SO (1.42) as compared to HCD alone, which again is consistent with increased oxidative metabolism.

### Triacylglycerol (TAG) Synthesis (Figure 7)

Increased glycerol and decreased glycerate are indicative of increased glycerol metabolism. Compared to chow, HCD (2.22), SO (2.97), and FO (1.57) increased levels of glycerol. Both HCD (0.57) and SO (0.72) decreased levels of glycerate (p<0.05), while FO did not.

**Figure 7:**
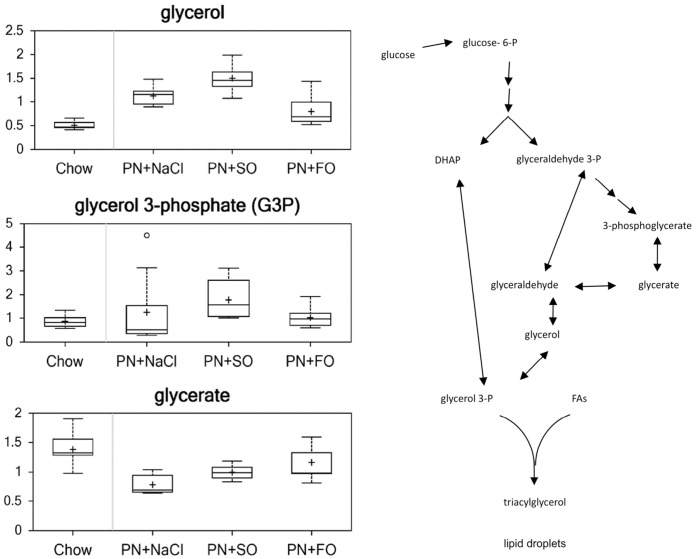
Metabolites of Triacylglycerol Synthesis.

Relative to chow, only SO raised glycerol 3-phosphate (G3P) levels (p<0.05), while HCD and FO did not. Relative to HCD, SO raised G3P levels (1.43; p<0.05), but FO did not (0.82). FO reduced glycerol (0.53) and G3P (0.57) relative to SO (p<0.05). HCD appears to increase lipogenesis, with these changes partially attenuated by FO but not SO.

### Phospholipids (Figure 8)

Phospholipids form lipid bilayers as part of cell membranes, and they are composed of three parts: a diglyceride, a phosphate group, and an organic molecule. Compared to chow, HCD, SO, and FO all had statistically significant reductions in phospholipid head groups – choline and ethanolamine– and their phosphorylated derivatives – choline phosphate and phosphoethanolamine (p<0.05). Most of the detected lysolipids, including 1-palmitoylglycerophosphocholine (0.42) and 1-stearoylglycerophosphocholine (0.39), showed significant decreases in HCD alone relative to chow (p<0.05). SO followed the same trend as HCD in most of lysolipids. However, FO reversed a significant number of changes observed in HCD alone and SO, including an increase in lysolipid levels close to that observed in the chow group. These changes are consistent with a normalization of phospholipid metabolism with FO administration compared to HCD and SO.

**Figure 8:**
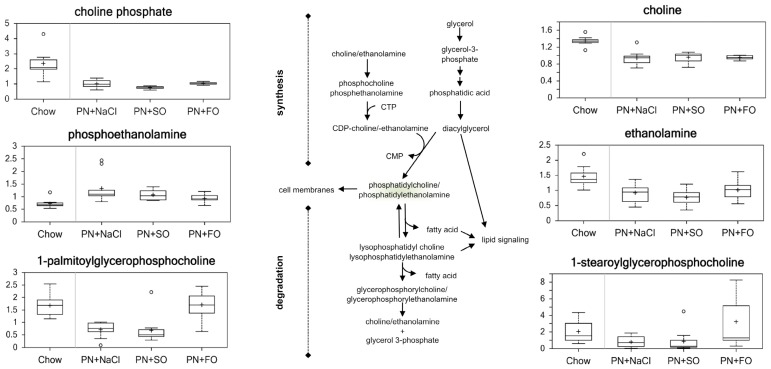
Phospholipid Metabolites.

### Other Membrane Lipid Components

Sphingolipids are a class of aliphatic amino alcohols that are key components of the cell membrane and important signaling mediators. The level of palmitoyl sphingomyelin increased significantly in HCD alone (1.62), SO (1.36), and FO (1.60) compared to chow (p<0.05). Compared to HCD alone, SO decreased levels of sphinganine (0.48) and sphingosine (0.42), while FO increased levels of sphinganine (2.57) and sphingosine (3.28) (p<0.05).

The level of hepatic cholesterol was significantly decreased in both HCD alone (0.82) and SO (0.87) groups relative to chow (p<0.05). FO had the same concentration of cholesterol as the chow group. Squalene decreased significantly in HCD alone relative to chow (0.35) and both SO and FO increased the level significantly above that in HCD alone (1.44 and 1.76 respectively, p<0.05). 7-α-Hydroxycholesterol, a precursor for bile acids derived from cholesterol, showed progressively significant decreases in the order of HCD alone (0.68), SO (0.40) and FO (0.26) (p<0.05).

### Additional Observations

Compared to SO, FO increased the levels of branched-chain amino acid (BCAA) catabolites, including 2-methylbutyroylcarnitine (1.78), isovalerylcartinine (1.84), hydroxyisovaleroyl carnitine (1.22), tiglyl carnitine (1.52), and methylglutaroylcarnitine (1.42) (p<0.05). These changes are consistent with either an increased or decreased use of branched chain amino acids for oxidation, which are primary fuels for skeletal muscle. The levels of riboflavin (1.47) and flavin adenine dinucleotide (1.23) increased in FO relative to SO, suggesting an improved capacity for energy production in response to FO supplement.

## Discussion

In this study, we sought to characterize global metabolic changes that occur in our mouse model of hepatic steatosis, with a focus on the effect of high-carbohydrate feeding and the differential effect of FO versus SO. Specifically, we focused on the metabolites that are critical to fatty acid synthesis and degradation, carbohydrate metabolism, triglyceride synthesis, inflammation, and oxidation. We confirmed our hypotheses: (1) HCD without lipid induced *de novo* fatty acid synthesis and oxidative stress; (2) Both FO and SO relieved some of the metabolic shift towards *de novo* lipogenesis; but (3) FO offered additional advantages in terms of lipid peroxidation and the generation of inflammatory precursors.

### Essential Fatty Acids and their Derivatives

The administration of HCD alone without any source of fat resulted in a significantly decreased concentration of the EFAs LA and ALA. This is expected given the absence of dietary LA and ALA, as well as the effect of insulin (stimulated by high glucose administration) to limit adipose tissue lipolysis and therefore contain endogenous LA and ALA. Except for DHA and DPA, the SO group increased all of the other measured FA relative to the HCD group, presumably related to the dietary presence of LA and ALA. However, SO supplementation resulted in a relatively lower degree of increase of the ω-3 FAs, consistent with the lipid composition that provides ALA, which only has limited conversion to EPA and virtually no conversion to DPA and DHA. [7]–[9].

Compared to all the other groups, FO supplementation resulted in significant increases in the ω-3 FAs and decreases in the ω-6 FAs, reflecting enrichment of very long chain polyunsaturated ω-3 FAs in FO emulsion. FO reduced the level of arachidonic acid and its derivatives (12-HETE and 15-HETE), as EPA is known to inhibit the production of arachidonic acid from LA as well as substitute for arachidonic acid in tissue membranes. [10], [11] Arachidonic acid, in particular, is well recognized as a potentially pro-inflammatory parent molecule whose metabolites serve a variety of functions in the physiologic response to tissue damage and stress. Broadly speaking, eicosaonids derived from arachidonic acid induce a pro-inflammatory phenotype, whereas eicosanoids derived from EPA and DHA promote the resolution of inflammation. ω-3 and ω-6 fatty acids are competitive substrates for the same set of desaturation and elongation enzymes. Therefore, the two types of FAs are competitive substrates and elevation of one type of FA will suppress the production of metabolic products from the other type of FA. This is principally by reducing its concentration in membrane phospholipids. ω-3 fatty acids, such as those in FO, suppress the production of AA-derived prostaglandins and leukotrienes, thereby shifting the physiologic homeostasis towards a less inflammatory state while also promoting an active resolution of inflammation through the production of resolvins, protectins, and maresins. [12], [13].

While the specific role of the multitude of eicosanoids in PNALD is unknown, inflammation is clearly a key part of PNALD pathogenesis. The administration of HCD in piglets has been associated with hepatic steatosis, insulin resistance, and hepatic inflammation, including increased myeloperoxidase activity and expression of proinflammatory cytokines and chemokines, particularly IL-2, CXCL2, TNFα, and IL-6. [14] Mice receiving HCD demonstrate histologic evidence of liver injury, including lobular inflammation, hepatocyte apoptosis, peliosis, and KC hypertrophy and hyperplasia. [15] Inflammation has a direct inhibitory effect on bile acid nuclear receptors, thereby inducing cholestasis, which is a hallmark of human PNALD. [16] Soybean oil (SO)-based lipid emulsions contain phytosterols – plant-derived steroid alcohols – that have been shown to impair bile drainage and induce hepatobiliary dysfunction. [17] The addition of ω-3 fatty acids to PN has been shown to directly suppress expression of inflammatory markers TNF-α and IL-6 and up-regulate anti-inflammatory IL-10, in addition to reducing hepatocyte inflammation through activated macrophages. [18] Hence, the provision of a lipid emulsion rich in ω-3 versus ω-6 fatty acids may be therapeutic in dampening the hepatic inflammatory response. Steatosis secondary to carbohydrate-induced *de novo* lipogenesis may increase the liver’s susceptibility to secondary insults from reactive oxygen species, endotoxins, and adipocytokines. [19].

### Oxidative Stress

An increased burden of reactive oxygen species, combined with the inability to detoxify these products, induces oxidative stress, which results in damage to cellular proteins, lipids, and nucleic acids. PN has been previously shown to increase free radical production, in addition to decreasing liver oxidative metabolism and enzymatic antioxidant defenses, potentially as a result of saturation of liver microsomal fatty acids. [20], [21].

There are several major cellular anti-oxidant pathways. Specifically, glutathione acts as an electron donor to reduce disulfide bonds formed between cysteine residues on cytoplasmic proteins. Glutathione is oxidized to glutathione disulfide (GSSG), which can then be converted back to reduced glutathione via glutathione reductase.

This metabolomics analysis suggests that HCD impaired glutathione metabolism. Supplementation with SO showed increased levels of reduced glutathione, but FO appeared to show the largest increase in reduced glutathione. Furthermore, there were several changes that indicated FO improved the antioxidant defense system. FO reduced the levels of oxidized products from linoleate (18∶2n6) –9 and 13-HODE, which are known to be inducers of reactive oxygen species. There was a marked increase in the antioxidant α-tocopherol in the FO group that reflects the underlying composition of the lipid emulsion. The concentration of α-tocopherol in the commercial FO emulsion (150–296 mg/L) is significantly higher than the concentration of α-tocopherol in the commercial SO emulsion (38 mg/L).

Our results support an improved redox state in animals supplemented with FO compared with SO. This finding is relevant for several reasons. Oxidative damage is thought to be critical in the development of PNALD, with evidence demonstrating that PN administration is associated with inhibition of the superoxide dismutase activity in hepatocytes, an increase of the malondialdehyde level, an increase in cytochrome c release from the mitochondria, and increased apoptosis. [22] The supplementation of glutathione in animals models reduces hepatic dysfunction. [23] FO is known to reduce superoxide dismutase and glutathione peroxidase activities as compared with a safflower oil emulsion. [24] Livers infused with FO have lower levels of glutathionyl 1,4-dihydroxynonenal adduct, indicating reduced oxidative stress. [25].

### Fatty Acid Synthesis

As noted in the histology, the administration of an oral HCD without lipid induced profound hepatic steatosis. SO also induced hepatic steatosis but to a lesser degree, while FO prevented the development of steatosis entirely. Our data revealed several distinct and simultaneous metabolic changes that are consistent with derangements in fatty acid synthesis in this mouse model.

The formation of malonyl-CoA is the committed step in FA biosynthesis. The level of malonylcarnitine normally reflects the level of malonyl-CoA and was significantly lower in the HCD group relative to the chow group, suggesting increased FA synthesis as a consequence of high glucose administration unimpeded by dietary ω-6 or ω-3 fatty acid provision that specifically limit *de novo* lipogenesis. [26], [27] EPA is known to inhibit hepatic *de novo* lipogenesis via sterol regulatory element binding protein 1-c. [28]–[30] This is also supported by the higher levels of saturated and monounsaturated LCFAs in the HCD group compared to the chow group. The source of this increase in FA synthesis was likely derived from increased carbohydrate flux through glycogenesis/glycolysis. This was coupled with enhanced lipogenesis through glucose-stimulated insulin secretion, which both fosters lipogenesis and prevents endogenous lipolysis to lower free fatty acid levels that would down regulate lipogenesis. The significant decrease in glycogen breakdown products (including maltotriose, maltotetraose, maltopentaose, and maltohexaose) in the HCD alone group in relation to the chow group indicates a lesser amount of glyogenolysis, although one would expect that glycogen synthesis would be dramatically increased by HCD leading to a net increase in stored glycogen. SO attenuated the increase in LCFA seen in HCD alone. However, SO only increased the level of malonylcarnitine slightly over that observed in HCD alone, indicating a small degree of moderation of the effect of HCD on FA oxidation. On the other hand, FO increased malonylcarnitine and decreased most of the free LCFAs significantly compared to both PN and SO groups, suggesting a marked reduction in FA synthesis and *de novo* lipogenesis as well as enhanced FA oxidation, consistent with the protective effect of FO on development of steatosis.

The intermediates in the TCA cycle increased significantly in both SO and FO groups in relation to HCD alone, indicative of an increased TCA cycle activity, more efficient energy production and less diversion of citrate for FA synthesis. Compared to the HCD alone or SO groups, levels of branched-chain amino acid (BCAA) catabolites in the FO group were significantly elevated and closer to that observed in the chow group. This observation may reflect a reduced demand for BCAA catabolites for FA synthesis in the FO group compared to the HCD alone and SO groups, and potentially reduced utilization of branched chain amino acids for oxidation by skeletal muscle.

### Triacylglycerol (TAG) Synthesis

TAG accumulation is a central feature of steatosis and is the result of disordered lipid metabolism, either in the form of increased TAG synthesis, defective TAG trafficking, or decreased TAG degradation primarily through oxidation.

Our analysis demonstrates that the HCD group had increased levels of glycerol and decreased levels of glycerate indicative of increased glycerol metabolism. This observation coupled with increased FA synthesis could result in increased TAG synthesis, observed histologically as the profound increase in lipid deposition. SO raised glycerol 3-phosphate levels, possibly offering some attenuation of HCD-induced increase in TAG synthesis. On the other hand, FO reduced glycerol, G3P and free LCFA levels significantly relative to the SO group. This observation could indicate a restoration of TAG synthesis to the normal level by FO and may be related to the protective effect of FO. This would be expected given the known ability of the ω-6 fatty acid LA and the ω-3 fatty acids ALA and EPA to reduce FA synthesis through effects on PPAR with the much greater impact of EPA. There were different priorities among the groups in cholesterol utilization; most notably, FO normalized hepatic cholesterol levels that were decreased in HCD alone and SO.

This mechanistic insight is supported by existing data on the effect of HCD on FA metabolism. HCD is high in simple carbohydrate content, and overconsumption of carbohydrates (particularly simple ones) enhances lipogenesis. The supplementation of HCD with lipid reduces hepatic triglyceride synthesis, enhances FA oxidation, and increases peripheral plasma triglyceride lipolysis. [31] FO is known to reduce hepatic triglyceride content, and the ω-3 derivative EPA has been shown to reduce the activity of acetyl coenzyme A carboxylase – the enzyme that catalyzes the irreversible carboxylation of acetyl-CoA to produce malonyl-CoA – and inhibit *de novo* lipogenesis. [32], [33].

### Limitations and Conclusions

There are several limitations to our study. This work is based on a mouse model of high glucose-induced hepatic steatosis, and therefore, the relevance to human PNALD is unknown. In our study, the HCD solution was administered orally to induce hepatic steatosis. Others have described an intravenous rat model of PNALD, but the duration of this model is short given the development of a high rate of sepsis in such animals. We felt that a longer duration of treatment would better simulate the metabolic changes of chronic PN dependence. Additionally, while this metabolomic approach represents a systematic and simultaneous analysis of a complex pathophysiologic state, it only provides a single snap shot, therefore limiting any conclusions about changes over time or the order of events, and a number of the changes are speculative based on static levels.

The present data detail the global metabolic consequences of PN and lipid administration, and suggest that improved lipid metabolism combined with reduced oxidative stress may explain the protective effect offered by FO supplement *in vivo*. Future work is necessary to determine how the manipulation of these pathways may have therapeutic benefit in PNALD.

## Acknowledgments

We would like to thank the team at Metabolon for their services in performing the metabolomic profiling, as well as their technical advice and analysis.
